# Space Radiation: The Number One Risk to Astronaut Health beyond Low Earth Orbit

**DOI:** 10.3390/life4030491

**Published:** 2014-09-11

**Authors:** Jeffery C. Chancellor, Graham B. I. Scott, Jeffrey P. Sutton

**Affiliations:** 1 National Space Biomedical Research Institute (NSBRI), and Center for Space Medicine, Baylor College of Medicine, 6500 Main Street, Suite 910, Houston, TX 77030-1402, USA; E-Mails: jeff.chancellor@bcm.edu (J.C.C.); graham.scott@bcm.edu (G.B.I.S.); 2 Department of Materials Science and Engineering, Dwight Look College of Engineering, Texas A&M University, 3003 TAMU, College Station, TX 77843-3003, USA; 3 Department of Molecular and Cellular Biology, Baylor College of Medicine, 6500 Main Street, Suite 910, Houston, TX 77030-1402, USA; 4 Department of Medicine, Baylor College of Medicine, 6500 Main Street, Suite 910, Houston, TX 77030-1402, USA

**Keywords:** space, radiation, radiobiology, omics, cancer, degenerative tissue effects, central nervous system effects, acute radiation syndrome, galactic cosmic radiation, solar particle events

## Abstract

Projecting a vision for space radiobiological research necessitates understanding the nature of the space radiation environment and how radiation risks influence mission planning, timelines and operational decisions. Exposure to space radiation increases the risks of astronauts developing cancer, experiencing central nervous system (CNS) decrements, exhibiting degenerative tissue effects or developing acute radiation syndrome. One or more of these deleterious health effects could develop during future multi-year space exploration missions beyond low Earth orbit (LEO). Shielding is an effective countermeasure against solar particle events (SPEs), but is ineffective in protecting crew members from the biological impacts of fast moving, highly-charged galactic cosmic radiation (GCR) nuclei. Astronauts traveling on a protracted voyage to Mars may be exposed to SPE radiation events, overlaid on a more predictable flux of GCR. Therefore, ground-based research studies employing model organisms seeking to accurately mimic the biological effects of the space radiation environment must concatenate exposures to both proton and heavy ion sources. New techniques in genomics, proteomics, metabolomics and other “omics” areas should also be intelligently employed and correlated with phenotypic observations. This approach will more precisely elucidate the effects of space radiation on human physiology and aid in developing personalized radiological countermeasures for astronauts.

## 1. Introduction

**Figure 1:**
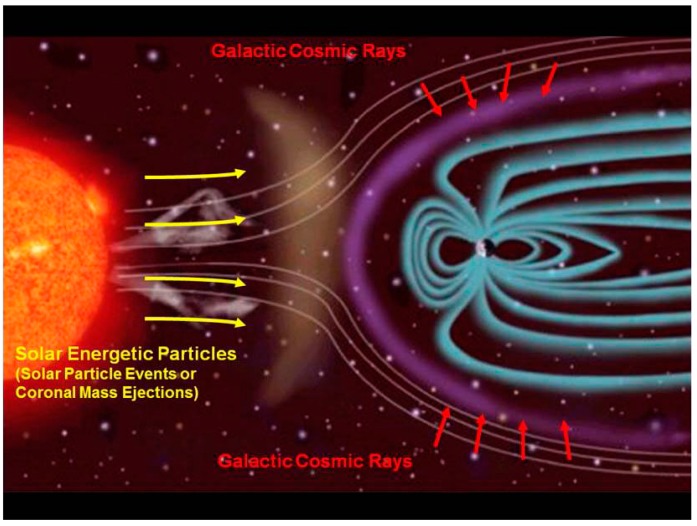
The interplanetary space environment showing the toxic combination of galactic cosmic radiation (GCR) and (largely) proton radiation due to solar particle events (SPEs). Figure courtesy of NASA/JPL-Caltech.

The space environment beyond low Earth orbit (LEO) contains several types of ionizing radiation. Most of the energetic particles found in interplanetary space are from the solar wind, which produces a constant flux of low linear energy transfer (LET) radiation. For missions outside of LEO, galactic cosmic radiation (GCR) will contribute a significant portion of the radiation dose accumulated by astronaut crew members. GCR ions originate from outside our solar system and contain mostly highly energetic protons and alpha particles, with a small component of high charge and energy (HZE) nuclei moving at relativistic speeds and energies [1]. In addition to GCR, unpredictable and intermittent solar particle events (SPEs) can produce large plasma clouds containing highly energetic protons and some heavy ions that may cause a rapid surge of radiation both outside and within a spacecraft (Figure 1).

Future human spaceflight missions potentially include Moon bases, rendezvous with a near-Earth object (NEO), such as an asteroid, and, eventually, habitations on the surface of Mars. For current space missions in LEO, the shielding provided by the Earth’s magnetic field attenuates the major biomedical effects of space radiation exposures. The risks of space radiation will, however, become more onerous, as future spaceflight missions to an NEO or Mars require extended transit beyond the protection of the Earth’s magnetosphere.

In 2006, the National Council on Radiation Protection and Measurements (NCRP) issued a report entitled, “Information Needed to Make Radiation Protection Recommendations for Space Missions Beyond Low-Earth Orbit” [2]. The report contains a comprehensive summary of the current evidence for radiation-induced health risks and makes recommendations on areas requiring further experimentation to enable future space missions beyond LEO. Specifically, the report states “Current space radiation guidelines pertain only to missions in LEO and are not considered relevant for missions beyond LEO. The acceptable levels of risk for space exploration beyond LEO have not been defined at this time and need to be dealt with before sending manned missions to colonize the moon or to deep space, such as a mission to Mars” [2]. Moreover, the NCRP report emphasizes the need for identifying and validating biomarkers for reliable early detection of adverse effects, improving radiation biodosimetry by providing accurate estimates of cumulative radiation doses and identifying increased personal risks for individual astronauts, due to genetic predisposition to the effects of space radiation.

Subsequently, in 2008, the National Research Council released another report “Managing Space Radiation Risk in the New Era of Space Exploration” [3]. The expert authors found that the “lack of knowledge about the biological effects of, and responses to, space radiation is the single most important factor limiting the prediction of radiation risk associated with human space exploration” [3].

Exposure to charged particles representing a wide array of atomic numbers, energies, dose rates and resulting secondary radiation cascades can induce health effects that are associated with both SPE and GCR exposures. The National Aeronautics and Space Administration (NASA) has identified four primary biomedical risks that may pose significant health concerns for astronaut crews exposed to the interplanetary radiation environment during exploration missions. These four space radiation risks are carcinogenesis, degenerative tissue effects, CNS decrements and acute radiation syndrome [4,5,6,7].

## 2. The Pernicious Interplanetary Space Radiation Environment

Radiation outside of LEO is composed of a toxic milieu of GCR and particles (predominantly protons) expelled from the Sun during SPEs. This mixture of radiation modalities represents the most significant physical impediment to safe human space exploration.

### 2.1. Galactic Cosmic Radiation

**Figure 2:**
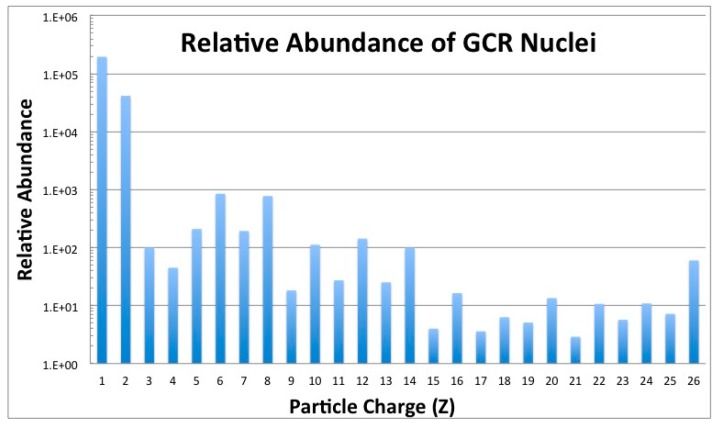
Relative abundance of GCR nuclei from hydrogen (Z = 1) to iron (Z = 26) [1].

GCR nuclei originate from outside our solar system and are high-LET relativistic particles, possessing sufficient energies to penetrate any shielding technology used on current mission vehicles [8]. The GCR spectrum consists of approximately 87% hydrogen ions (protons) and 12% helium ions (alpha particles), with the remaining 1%–2% of particles being HZE nuclei with charges ranging from Z = 3 (lithium) to approximately Z = 28 (nickel) [9]. Ionized transition metals, such as iron (Z = 26), are biologically harmful, as no reasonable amount of spacecraft material can shield them (Figure 2). Electrons and positrons make up about 1% of the overall GCR spectrum, but are considered a minor biological hazard, since they are easily stopped by even a modest amount of spacecraft shielding.

The fluence of ionized nuclei that make up GCR is inversely proportional to the solar cycle and decreases by a factor of two during solar maximum [10]. The GCR fluence rate and spectrum outside of LEO have been generally characterized through measurements made by unmanned spacecraft, such as the Mars Science Laboratory (MSL) spacecraft that, over the period December 2011 through July 2012, carried the Mars Curiosity rover to the red planet [11]. Recent evidence has therefore demonstrated that the absorbed dose and dose equivalent from incident particles can be well estimated in advance of future exploration class space missions.

The large ionization power of GCR ions makes them a major health threat to astronauts and constitutes one of the most important barriers impeding plans for interplanetary travel by crewed spacecraft. GCR particle energies are sufficient to penetrate several centimeters of biological tissue or other organic and inorganic materials. Shielding only partially reduces the doses experienced inside a spacecraft, given the penetrating ability of HZE ions [8]. While thicker shielding could in theory provide more protection, deploying a sufficient mass of shielding into space is limited by the practical capabilities of current spacecraft launch systems.

During transit outside of LEO, every cell nucleus within an astronaut would be traversed, on average, by a hydrogen ion every few days and by heavier HZE nuclei (e.g., ^16^O, ^28^Si, ^56^Fe) every few months [12]. Therefore, in spite of their low flux, HZE ions constitute a deleterious biological threat and contribute a significant amount to the cumulative GCR dose that astronauts will incur outside of LEO.

### 2.2. SPE Radiation

**Figure 3:**
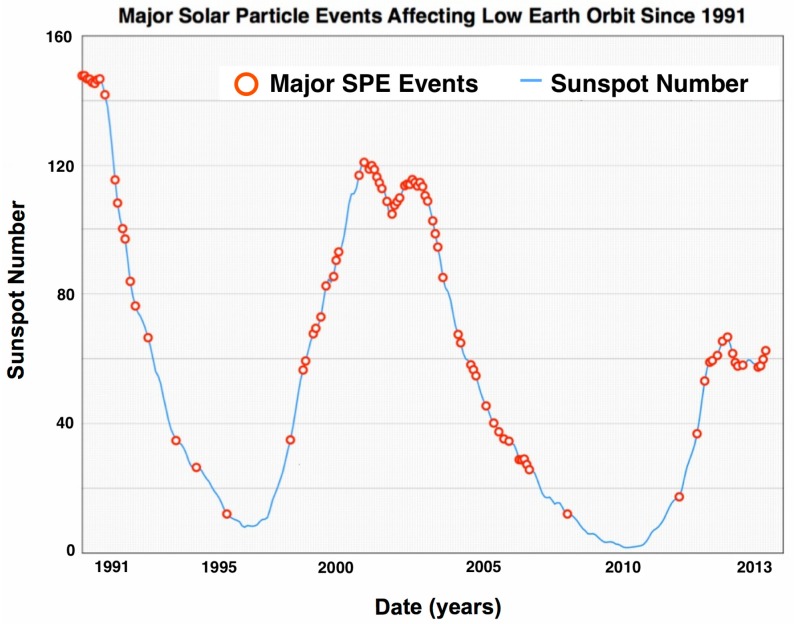
Energetic SPEs affecting low Earth orbit (LEO) space missions since 1991 are plotted as a function of the solar cycle. Shown here are events (red circles) that have been measured since 1991 to 2013 and include Solar Cycle 22 (partially), 23 and 24 (partially). Energetic solar events contain a higher fluence of >100 MeV protons that can penetrate typical spacecraft shielding and significantly impact the health of astronauts.

Dangerous and unpredictable SPEs can produce large quantities of energetic protons with fluences in excess of 10^9^ protons/cm^2^ [5]. SPEs consist largely of low-LET protons with energies ranging up to 1 GeV/n that can be shielded relatively easily by spacecraft hulls. Even so, there can be very high density fluxes of protons with energies greater than 30 MeV that can be of concern to astronauts in thinly-shielded vehicles and habitats [13,14]. SPE dose rates are variable over the course of an event and range from zero to 100 mGy/h inside a space vehicle and from zero to 500 mGy/h for an astronaut exposed during an extravehicular activity (EVA) on missions outside of LEO [6]. The frequency of SPEs is proportional to sunspot activity, and SPE occurrences wax and wane with the phase of the 11-year solar cycle, peaking when equatorial sunspot activity is highest. The phase of the solar cycle, however, does not determine the intensity of SPEs, and some of the largest measured solar events have occurred during off-peak periods, when there has been a significant reduction of observed sunspots (Figure 3).

Exploration missions outside of LEO will include interplanetary transits, and shielding provided by the spacecraft may not be able to completely protect astronaut crews from the effects of an SPE. Furthermore, it is likely that crewmembers will be exposed to multiple SPEs during such missions. For example, five SPE events were recorded during the recent transit of the MSL spacecraft from Earth to Mars [11].

### 2.3. Intravehicular Radiation

The interaction of energetic SPE protons and heavy-charged GCR particles with the spacecraft structure can produce an additional, secondary intravehicular radiation hazard. Secondary particles produced in nuclear fission reactions include protons, alpha particles, beta particles, gamma rays, x-rays, neutrons and heavy-charged particles. Created by passing through spacecraft shielding, these fission products can deliver a significant fraction of the total mission dose and have the ability to damage critical cellular components when passing through the tissues of the body.

A very small amount of radionuclides are used by spacecraft instrumentation, but the majority of crew exposures are due to the complex external radiation environment.

## 3. Biomedical Consequences of Exposure to Space Radiation

It remains unclear how moderate to large magnitude SPEs, when combined with continuous GCR exposure, will affect the health and performance of astronaut crews during interplanetary transits.

SPEs have a unique dose distribution with respect to whole body irradiation. For instance, skin doses are 5–10-times higher than those experienced by internal organs, because of its superficial location and susceptibility to absorption in the low energy spectra of protons and nuclei [15]. SPE radiation and the synergistic effects of spaceflight can place the crew at significant risk for prodromal effects (e.g., nausea and vomiting), skin injury, hematological changes and immune system dysfunction. The current assessment is that the risk of death is low as a result of a major solar event or the combined effect of multiple SPEs. A similar claim pertains to the accumulated GCR exposure over the course of exploration class missions to Mars, the Moon or an asteroid.

However, the morbidity risk and unique toxicity profiles remain poorly understood. This incomplete understanding persists, despite the existence of a significant body of literature describing the effects from anticipated absorbed dose ranges. Calculation of radiation exposures to astronauts in a detailed and realistic way is challenging because of the complexity of the radiation environment, the shielding effects of the vehicle and/or space suit and human anatomy and physiology. Uncertainties with respect to dose toxicity and the complex variation in SPE and GCR spectra likely to be encountered in future exploration missions underscore the need for models that are capable of identifying particle energy and species on an event-by-event basis. Additionally, integrating microdosimetry measurements with radiobiological studies are essential to reducing the uncertainties in dose projections during mission planning, spaceflights and to inform post-flight research on astronaut health.

**Figure 4:**
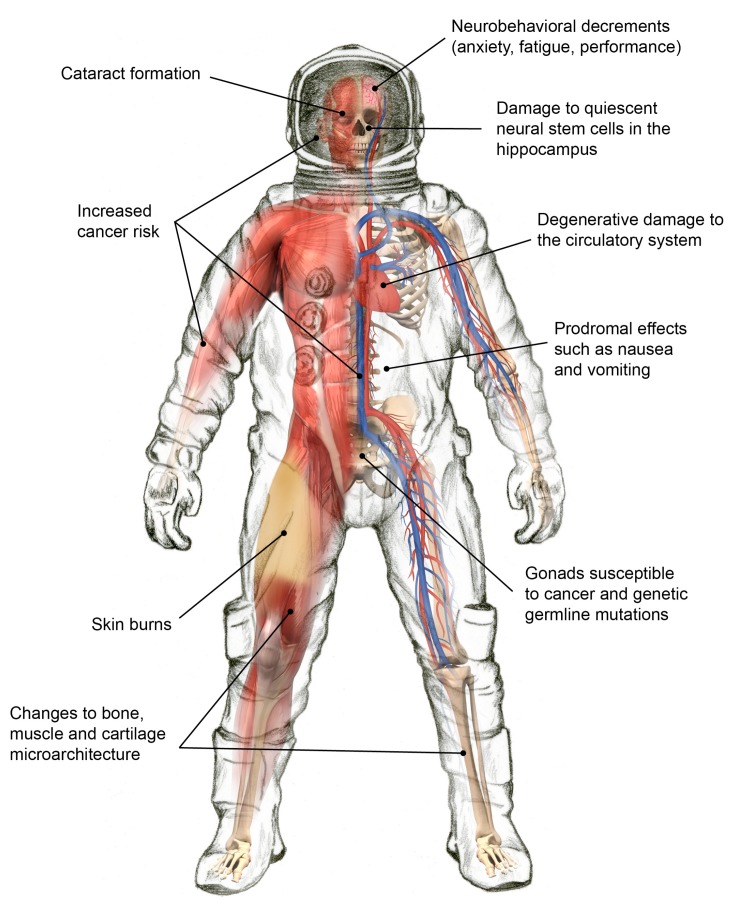
Select health effects due to space radiation exposures.

Exposure to space radiation affects multiple organs and physiological systems in complex ways (Figure 4). NASA categorizes the biomedical consequences into four risk areas. Each is discussed in the remainder of this section.

### 3.1. Degenerative Tissue Effects from Radiation Exposure

The main radiation health risks to astronaut crews on exploration missions are degenerative vascular changes, genetic mutations and cancer [2]. While the International Space Station (ISS) has been continuously crewed for fifteen years, with each astronaut spending an average of six months in space, there are limited data to evaluate the degenerative tissue effects that may arise due to a high radiation dose delivered over several months [16].

To address the non-cancerous, late effects of radiation appearing months or years after exposure, the authors of the 2006 NCRP Report recommended that experiments be conducted using protracted or extended exposure times and low dose rates of protons, heavy ions and neutrons in energy ranges that are relevant to the space radiation environment outside of LEO. Specifically, the authors of the report recommended that analyses be conducted to study the effects of protracted radiation exposures on the lens of the eye, whole-body vasculature, gastrointestinal tract, gonadal cell populations, hematopoietic and immune systems and fertility.

With respect to the different qualities of space radiation that may be encountered, the high LET radiation found in the GCR spectrum can directly or indirectly damage biomolecules (e.g., proteins, DNA, lipids), as well as organelles and cellular structures. The resultant radiation-induced increase in oxidative stress levels has been shown to exacerbate some degenerative tissue changes that are normally associated with aging (e.g., cardiovascular disease and cataract formation). In addition, it is thought that certain aspects of spaceflight, such as microgravity, as well as the artificial and confined environment may accentuate degenerative tissue responses. No studies to date have adequately assessed the possibility of synergism between high-LET radiation and the stresses of the space environment.

Epidemiological studies on atomic bomb survivors in Japan, radiotherapy patients and occupationally-exposed workers have characterized the association between moderate to high doses of low-LET radiation and the long-term development of degenerative tissue effects, such as heart disease, cataracts, immunological changes and premature aging [17,18,19,20]. These findings are supported by laboratory studies using animal models [21,22,23,24,25,26]. However, the risks for these same effects occurring after low dose rate or HZE nuclei exposures are more difficult to assess, due to the multifactorial nature of the diseases and their long latency periods. Furthermore, there is only a small probability that low-LET radiation from SPEs will reach high enough doses to cause degenerative tissue effects. It also remains unclear whether low-dose (*i.e.*, <0.5 Gy) exposures influence the same biological mechanisms that have been shown to be involved in disease progression following moderate- to high-dose SPE radiation exposures. Likewise, as with high-LET radiation, little information is available regarding the role of individual susceptibilities and possible synergistic effects with other spaceflight factors [7].

The 2006 NCRP Report directly addressed the utility of genomic, proteomic and, by extension, potentially other omics studies to elucidate the effects of space radiation on living systems. Specifically, the authors of this report stated, “It has been suggested that genetic screening of individuals for evidence of radiosensitive genes may become an important future criteria for selection of candidates for missions beyond LEO.” This report went on to explain, “The study of space radiation effects on various tissues of the body has revealed a previously unappreciated role for low-dose tissue remodeling involving stromal cell populations as well as cytoskeletal rearrangements in individual cells. These epigenetic effects involve changes in protein expression independent of the rapidly expanding work on direct radiation effects on gene expression. What is clear is that a different complement of genes and phosphorylated proteins is activated by exposure to low doses of conventional radiations, compared to the complement activated by higher doses of radiation. The ultimate medical consequences of perturbations in both genetic and epigenetic endpoints is, however, completely unknown. The radio-sensitivity of tissue-specific stem cells and endothelial cells remains a concern” [2].

The linkage between DNA lesions (including point mutations, insertions and deletions, as well as intra- and inter-chromosomal rearrangements) and carcinogenesis is well-established [27]. What is far less clear is whether genetic mutations are also implicated in late or degenerative radiation effects, such as circulatory system decrements. To date, there has been little research relating DNA genetic and epigenetic damage to degenerative radiation-induced tissue effects at the low doses associated with the space environment found outside of LEO.

Cytogenetic data unequivocally reveals that space radiation exposure produces significant damage to cells [28]. Indeed, post-flight chromosomal breaks were first observed using the Giemsa staining technique in the Gemini and Apollo astronauts during the 1960s and early 1970s. This work showed that chromosome breaks were two-fold more frequent in the Apollo astronauts compared to the Gemini astronauts, suggesting for the first time a link between dose and flight duration. Interestingly, the Apollo and Gemini data also showed some inter-individual differences. In hindsight, these findings are not surprising, given the known heterogeneous responses to radiation following even standardized irradiation protocols. Age, sex and immune status are all factors that affect responses to ionizing radiation. Moreover, there are other parameters germane to the space environment, such as microgravity, habitat control and stress.

The ISS was launched in 1998, allowing for the collection of biomolecular data that further informed the response of the human body to space radiation. Additional conclusions could be drawn about the fate of the chromosomal aberrations at time points long after flight and between successive flights. This included the intriguing observation that the yield of chromosomal aberrations decreases some years after a first flight, but without reaching the un-irradiated values. Moreover, a second spaceflight apparently does not proportionately increase the yield of aberrations, suggesting a non-additive or even an infra-additive effect, raising the possibility of a radio-adaptive response in crewmembers (*i.e.*, “radiation hormesis”).

**Figure 5:**
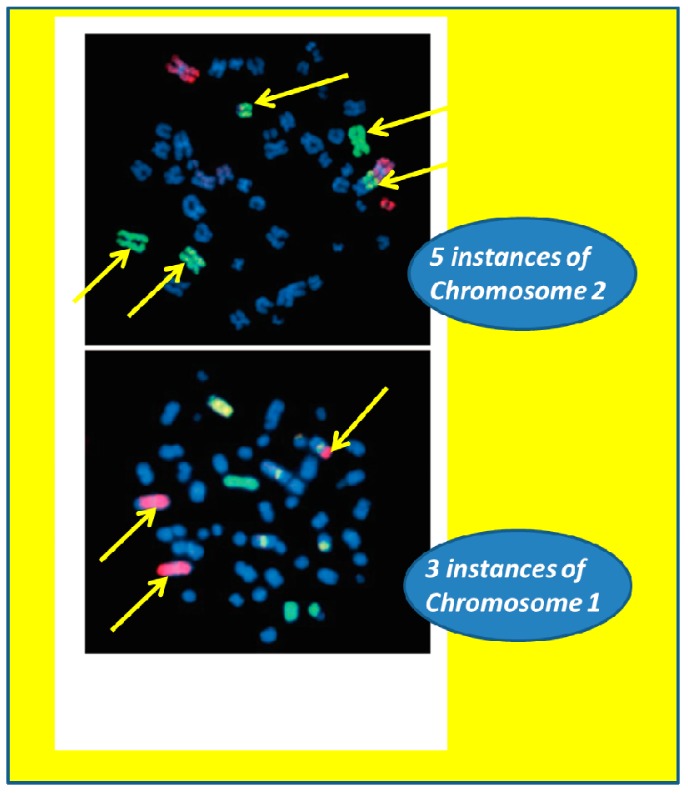
Examples of two complex aberrations involving three or more chromosomes observed post-mission in astronauts. Chromosomes were hybridized with painting probes for chromosome 1 (red), chromosome 2 (green) and chromosome 4 (yellow). All other chromosomes were counterstained with DAPI (blue). Adapted from Cucinotta *et al.* [29] and republished with permission from *Radiation Research*.

In 2008, using multi-color fluorescence *in situ* hybridization, Cucinotta *et al.* vividly showed complex chromosomal aberrations in lymphocyte cells involving three or more chromosomes, observed post-mission in ISS astronauts [29], (Figure 5). This work was significant, as it demonstrated gross biomolecular damage at the fundamental DNA level within ISS crewmembers as a result of exposure to space radiation [29].

Notwithstanding this finding, knowledge of the basic mechanisms specific to low-dose radiation, to sequential doses of low-dose radiation and to adaptive responses is still at a rudimentary stage. Experiments utilizing new and more powerful “omics” approaches (e.g., genomics, transcriptomics, proteomics, metagenomics, metabolomics and epigenomics) that probe genomic instability and delayed mutagenesis, when tightly correlated with phenotype, may prove to be extremely helpful in quantifying the risks of potential space radiation-induced degenerative diseases.

### 3.2. Radiation Carcinogenesis

It still remains to be determined whether the higher doses of radiation incurred during future exploration class missions beyond LEO will increase the threat of cancer for astronaut crews [4]. The elevated risk for astronauts developing cancer during or following a mission is directly related to the dose of radiation received [30]. Shavers *et al.* determined that the doses received by astronauts during extended ISS missions were typically greater than 70 mSv [31]. Even though it has been well understood that exposure to these and higher doses of radiation can be carcinogenic [30], most of the data available come from epidemiology studies of Japanese atomic bomb survivors, as well as research employing animal and cell models. These types of studies typically utilized both high dose rates and gamma exposures [3]. There have been no studies conclusively demonstrating that exposures to the unique doses and dose rates found in the space radiation environment will result in an increased risk of cancer mortality when compared to the U.S. population [32,33,34,35,36]. This uncertainty is due to several confounding factors, including: (1) the relatively low doses and dose rates of radiation received during spaceflight in LEO; (2) a wide range of time intervals between spaceflight and the current ages of individual living astronauts; and (3) the small overall sample size of those who have flown in space. These complicating factors collectively limit robust statistical confirmation [16].

A better understanding of the underlying molecular mechanisms is still a critical need in quantifying and elucidating the risk of space radiation-induced carcinogenesis. Research efforts focused on studying chromosomal aberrations in astronauts and cosmonauts have been conducted for risk assessment after single and multiple flights. However, interpretation of these studies is limited by significant individual variability and insufficient statistical power [37,38,39,40]. This has led to the dose-limiting guidelines that are currently amongst the highest occupational radiation exposure levels [29], (Table 1).

**Table 1:** Example career effective dose limits in units of Sieverts as calculated for one-year ISS missions. The radiation exposure-induced average life-loss per death for carcinogenesis is shown in parenthesis [41].

Age in Years	Dose Limit-Male Astronauts (Average Life-Loss Per Death in Years)	Dose Limit-Female Astronauts (Average Life-Loss Per Death in Years)
25	520 mSv (15.7)	370 mSv (15.9)
30	620 mSv (15.4)	470 mSv (15.7)
35	720 mSv (15.0)	550 mSv (15.3)
40	800 mSv (14.2)	620 mSv (14.7)
45	950 mSv (13.5)	750 mSv (14.0)
50	1150 mSv (12.5)	920 mSv (13.2)
55	1470 mSv (11.5)	1120 mSv (12.2)

### 3.3. Acute and Late CNS Effects from Radiation Exposure

Ionizing radiation can damage the CNS, causing changes to cognitive function, inducing fatigue, and generating performance decrements [2,30,42,43,44,45,46,47,48,49,50,51]. However, it is still unclear how SPE, GCR and constituent HZE nuclei negatively impact the CNS at the dose and dose rates found in the space environment [2,52,53]. Highly ionizing HZE nuclei can easily penetrate space vehicle structures and still have sufficient energy capable of heavily damaging cells or creating micro-lesions along their tracks through tissues [54].

Recent studies at NASA’s Space Radiation Laboratory using heavy-ion beams to simulate the GCR environment have provided evidence of the CNS health risk for missions outside of LEO. Britten *et al.* have shown that doses as low as 20 cGy of simulated GCR radiation (1 GeV/u ^56^Fe particles) can significantly impair learning and memory in a rodent model. These results demonstrate that mission-relevant doses of HZE particles may result in deficits in hippocampus-dependent neurocognitive tasks, most likely due to the perturbation of multiple processes, in addition to killing neuronal cells [55,56].

Further research has shown that similar doses result in the loss of functionality in several regions of the cortex, namely the medial prefrontal cortex, anterior cingulate, posterior cingulate and basal forebrain. These findings raise the possibility that astronauts on prolonged deep space exploratory missions could develop deficits in executive function [56]. Understanding the mechanisms is complicated, as Rabin *et al.* have shown that studies modelling the impact of HZE particles on cognitive performance cannot be generalized from radiobiology studies of chromosomal aberrations and carcinogenesis [57].

Space radiation-induced CNS effects are not limited to the effects of HZE particles. Hienz *et al.* demonstrated that proton radiation caused marked neurocognitive deficits at doses as low as 25 cGy. These dosages are significantly lower than those associated with detrimental CNS changes seen in some cancer patients being treated with clinical radiotherapy, typically at doses of 25–50 Gy. Such doses are well above the predicted mission relevant exposures for missions outside of LEO.

In the studies by Hienz and co-workers, the psychomotor vigilance test [58,59], used by astronauts on the ISS to measure alertness levels, was adapted to assess motor and cognitive functions in a rat model. Susceptibility to radiation-induced CNS changes was measured, and it was shown that the more radiosensitive animals exhibited significant changes in proteins associated with dopamine receptors and transporters in the brain. These results may indicate that dopamine levels have an important role in neurobehavioral response to radiation [60,61].

These research data demonstrate that important changes to the CNS can be expected to occur at mission-relevant dose and dose rate levels. Research is still needed to clearly elucidate the significance to astronaut morbidity. Furthermore, the interpretation of animal-based CNS studies that model the space radiation environment is limited and confounds the development of mitigation strategies and countermeasures.

### 3.4. Radiation Syndromes Due to SPEs

During long-duration exploration missions beyond LEO, it is anticipated that multiple SPEs will be encountered. Shielding by the spacecraft or surface habitat would most likely be modest, and there is the threat of a single large SPE that could occur during an EVA. It is only during large SPEs (e.g., similar to the August 1972, SPE event) that the dose rate will rise to levels known to have substantive biological effects [62].

Kennedy *et al.* utilized mouse, ferret and mini-pig models to calculate blood cell and immune system parameters, as well as skin effects, for high dose rate SPE radiation [63,64]. In the mini-pig, it was demonstrated that high doses of SPE-like radiation resulted in adverse effects to the skin, as well as to deeper organs and tissues (e.g., decrease in circulating blood cells [63], lung damage [63] and impaired heart function [64]). Immune system suppression occurred at doses as low as 1.0 Gy. In a mouse model, T-cells did not become activated following exposure to low doses of SPE-like proton radiation, and the animals were susceptible to a non-toxic bacterial challenge of organisms (*Pseudomonas aeruginosa*, *Klebsiella pneumoniae*) that are relatively innocuous and ubiquitous on Earth and have been detected aboard the ISS [65,66].

**Figure 6:**
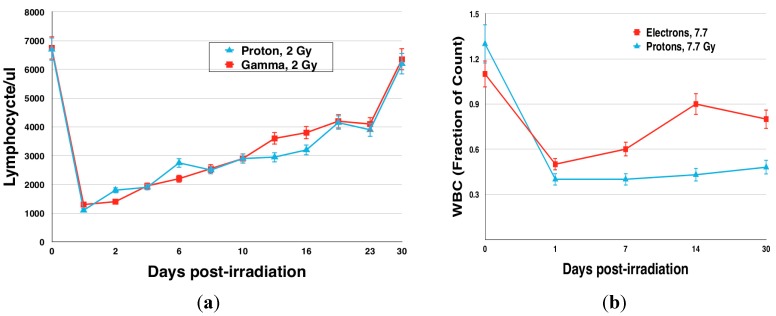
Acute radiation outcomes: (**a**) Blood cell counts (lymphocytes) following exposure to SPE-like gamma and proton radiation in a mouse model. Graph adapted from Romero-Weaver *et al.* [69]. (**b**) WBC counts in a mini-pig model following exposure to SPE-like electron and proton radiation. The WBC counts did not return to normal levels at the 30-day time point, with proton radiation exposure having the more detrimental effect. Graph adapted from Ann Kennedy [70] and reproduced with permission from *Life Sciences in Space Research*.

Furthermore, in the mouse model, high dose rate proton exposures resulted in comparable effects on the number of circulating white blood cells that subsequently returned to pre-irradiation levels within 30 days following exposure. In contrast, the blood cell counts (e.g., platelets, lymphocytes, neutrophils) did not return to normal, pre-irradiation levels in the mini-pig model following exposure to simulated SPE radiation. These results indicate that the mini-pigs, and other larger species, may be relatively incapable of repairing DNA damage caused by the proton radiation exposure as efficiently as rodents (Figure 6).

Astronauts performing EVAs have compromised shielding that can leave them vulnerable to the effects of unpredictable SPEs. Emesis and retching are known prodromal outcomes following exposures to high doses of radiation and can be detrimental, especially in the confined environment of an EVA suit. Animal studies performed using a ferret model indicate that retching and vomiting occur at doses as low as 50 cGy and can be expected from SPE radiation exposure at doses up to 2 Gy [67,68].

Fatigue is also thought to be a likely adverse effect of SPE exposures, and it has been observed in radiotherapy patients receiving therapeutic radiation doses as low as 2 Gy. Using a mouse model, studies suggest that exposure to low-dose rate SPE-like proton radiation leads to an increase in fatigue [71,72]. 

## 4. Synergistic Effects and Individual Susceptibility

Results from animal studies examining the synergistic effects of radiation combined with spaceflight environment stressors (*i.e*., microgravity, environmental constraints, emotional stress) show increased susceptibility to infection, delayed wound healing and decreased survival [73,74,75]. There are medical countermeasures available for the management of different acute injuries (e.g., burn care, wound closure and treatment, trauma minimization and infection control); however, only limited testing has occurred to study the efficacy of these measures and pharmaceuticals when radiation exposure is a concomitant and aggravating factor. There is a need to understand the mechanisms behind the synergistic lethality observed with radiation injury. It is also necessary to define appropriate animal models to determine the efficacy of treatments against damage resulting from radiation when combined with injury and to identify appropriate targets for the development of novel countermeasures [76].

Previous radiobiology research has shown individual differences in responses to radiation at all levels (*i.e.*, molecular, cellular and tissue) of biological organization. The difference in sensitivity has been linked to specific genetic variants implicated in some genetic diseases. However, it still remains unclear how these responses will relate to radiation-induced acute effects and carcinogenesis. Despite recent advances in integrated omics methodologies, genomic sequencing and a growing list of genes implicated with radiation sensitive phenotypes, no direct connections can yet be drawn between observed DNA sequence changes and a corresponding change in radio-sensitivity [27].

## 5. Research Considerations

There are many considerations to advancing knowledge about and developing operationally feasible countermeasures to combat the harmful effects of space radiation. Among the highest priority considerations are the appropriate simulation of the space environment for Earth-based studies and the selection of relevant animal models.

### 5.1. Simulating the Space Radiation Environment

In studies involving cells, tissues and model organisms conducted over many decades, radiobiologists typically have investigated exposures to proton and heavy ion sources separately. While this compartmentalized approach to space radiation research has been productive, it also represents a shortcoming in experimental design that is only now being addressed through concatenated and integrated studies addressing both the SPE and GCR effects [77].

Whether focusing on SPE- and GCR-like radiation, there is growing appreciation of the usefulness and limitations of the LET concept in describing a biological effect [78]. The LET of an ionizing particle describes the interaction of radiation with tissue matter and is the basis for determining the radiation quality of differing ionizing particles [10]. However, LET does not adequately describe in a single term all of the features of radiation quality. Two ionizing particles of different atomic numbers having the same LET can have very different track structures and fragmentation contributions.

An additional challenge is that modelling the GCR spectrum, dose and dose rate is complicated. Currently, mono-energetic beams (e.g., ^3^Li, ^12^C, ^28^Si, ^56^Fe) are utilized at research facilities, and the projected doses for a Mars mission are given using one or more acute heavy ion exposures. This method of delivering the heavy ion radiation in a single acute dose, rather than titrating it in a series of smaller chronic doses delivered over time, does not accurately model low-dose-rate effects. These include the impact on cell growth and the initiation of repair mechanisms. Similarly, radiobiological models and experiments utilizing mono-energetic beams of protons for short periods of time may not fully characterize SPE effects and the associated acute and sub-acute biological responses.

Thus, there are challenges to simulating the space radiation environment and effects. This is tempered by evidence that space radiation exposures at the dose rates expected for a mission to Mars may not induce significant acute or sub-acute biological responses. Nevertheless, there is a gap in knowledge given the limitations in obtaining realistic biological responses to simulated SPE and GCR exposures in the laboratory. More work in this important area is needed to improve our understanding.

### 5.2. Selection of Appropriate Animal Models

In 2004, the National Institute of Allergy and Infectious Diseases sponsored a workshop entitled, “Animal Models for Radiation Injury, Protection and Therapy”. The main goals of the workshop were to identify the most appropriate animal models to evaluate radio-protectors and therapeutic agents (including both preventative measures and post-exposure treatments), to develop accurate and user-friendly biodosimetry methods and to identify gaps in the research infrastructure needed to advance mechanistic studies and product development for protection against, mitigation of and treatment of radiation injury [79].

The group concluded that animal models are applicable to the following two stages of radiation research: (1) mechanisms/discovery; and (2) validation. Each stage requires different animal models. In some instances, it is also possible to investigate radiation therapy patients for validation of the efficacy of protectors, preventative measures and therapeutic agents.

Animal models are utilized in radiobiology studies as surrogates for humans. In general, animal experiments have contributed significantly to our understanding of the mechanisms of disease, but their value in predicting the effectiveness of treatment strategies has limitations [80,81,82]. Using animal models to study the effects of space radiation can generate results that are often difficult to interpret. Some commonly-used animal models do not accurately reflect the pathologies and pathologic response (*i.e.*, outcomes and endpoints) seen in humans, and disparities and genetic differences can also contribute to the difficulty in translating countermeasures into clinical use. These obvious physiological and genetic differences, combined with response-specific disparities between model species (e.g., rodent, porcine, primate), make it difficult to translate research results into clinical outcomes. In particular, the threshold doses required to generate specific measurable physiological effects can be very different between species and even between strains within species [83]. Additionally, the selection of a single animal model for specific organ system studies may prove inadequate due to physiological interactions between organ systems that modify specific radiation toxicities.

Therefore, it is critical to utilize a variety of appropriate animal models for the discovery, validation and eventual clinical utilization of novel biomarkers and countermeasures relevant to protecting astronaut crews from radiation on extended missions beyond LEO.

## 6. Conclusions

Space radiation is the number one risk to astronaut health on extended space exploration missions beyond the Earth’s magnetosphere. Only 24 human beings have ventured beyond this protective envelope, and then, only for a maximum of approximately 12 days (Apollo 17). This represents a vanishingly short amount of time that humans have spent in the interplanetary radiation medium, certainly relative to the multi-year timeframe for a mission to the Mars.

As humankind prepares to embark on increasingly ambitious and potentially dangerous deep space missions, considerable detailed work is required to better characterize and mitigate, to an acceptable level, the risks associated with space radiation exposures. The veracity of data obtained and the reliability of conclusions drawn from ground-based studies using model organisms will benefit substantially from accurately simulating combined exposures to both protons and heavy ions sources. These need to be delivered in a low-dose regimen and not delivered in a single acute dose. Furthermore, omics techniques should be utilized in an integrated fashion to complement and more completely inform physiological endpoint observations.

The ability of humans to realize our potential by becoming a multi-planet species will hinge on, to a large extent, our ability to appropriately understand, manage, mitigate and overcome the significant dangers and health effects of space radiation.
